# Xerostomia in primary care: a register-based study of prevalence, medication categories, and associated risk factors

**DOI:** 10.3389/froh.2025.1684568

**Published:** 2025-10-10

**Authors:** Vini Rughwani, Junmei Miao Jonasson, Bertil Marklund, Karin Mossberg, Annica Almståhl, Anne Marie Lynge Pedersen, Hülya Cevik-Aras

**Affiliations:** 1 School of Public Health and Community Medicine, Institute of Medicine, Sahlgrenska Academy, University of Gothenburg, Gothenburg, Sweden; 2 Department of Oral Microbiology and Immunology, Institute of Odontology, Sahlgrenska Academy, University of Gothenburg, Gothenburg, Sweden; 3 Section for Oral Biology and Immunopathology/Oral Medicine, Department of Odontology, Faculty of Health and Medical Sciences, University of Copenhagen, Copenhagen, Denmark; 4 Department of Oral Medicine and Pathology, Institute of Odontology, University of Gothenburg, Gothenburg, Sweden

**Keywords:** dry mouth, drug, polypharmacy, healthcare, diagnosis, primary care, ATC classification, Sjögren's disease

## Abstract

**Objectives:**

Xerostomia, the subjective sensation of oral dryness, is often associated with reduced salivary secretion. Xerostomia is a prevalent side effect to numerous medications but also found associated with aging and female sex. The objective of the study is to estimate the prevalence of xerostomia in association with the number and type of medications used by adults in the region of Västra Götaland, Sweden.

**Materials and methods:**

Data on age, sex, and medication use in patients diagnosed with xerostomia, using International Classification of Disease (ICD) 10 code 11.7 and R68.2 was obtained from the health care database in the region of Västra Götaland, Sweden (Vega). Prevalence of xerostomia diagnosis was estimated according to the type and number of medications consumed, stratified by age and sex.

**Results:**

The overall prevalence of xerostomia diagnosis was 0.23%. Medication was strongly associated with xerostomia, with the highest percentage of diagnosis observed in patients taking medications for treatment of metabolic diseases and diseases in alimentary tract (16.07%), the nervous system (16.04%), and the cardiovascular system (12.63%). Xerostomia was also associated with the number of medications. Polypharmacy (concomitant intake of five or more medications) and aged over 71 years was associated with 9.68-times higher odds for xerostomia (*p* < 0.0001). Females were more likely to be diagnosed with xerostomia, representing 73.08% of cases compared to 26.92% for males (*p* < 0.0001). The prevalence of xerostomia was lowest in the 18–35 age group (9.56%) and highest in those aged above 71 years (41.49%). Patients aged 55 years and older were significantly more likely to be diagnosed with xerostomia than younger patients (*p* < 0.0001).

**Conclusions:**

The findings emphasize a strong association between medication use, particularly polypharmacy, and xerostomia, with substantial variations across age, sex, and medication categories. Despite the high risk, a low prevalence of xerostomia diagnosis was reported in primary care settings. The findings suggest a need for increased clinical awareness and routine assessment to improve detection and management.

## Introduction

Xerostomia is the subjective experience of having a dry mouth (1) and this symptom is typically but not always associated with salivary gland hypofunction (hyposalivation) (2, 3). Xerostomia has often negative impacts on the quality of life by contributing to oral and psychosocial consequences (4, 5). Individuals suffering from oral dryness, particularly those with hyposalivation, often experience difficulties with eating, swallowing, speech, and taste alterations (6, 7). Moreover, increased tooth decay, taste disturbances, burning sensation in the mouth, and fungal infections are also common problems (8, 9).

Xerostomia can be assessed using a single-item question “Have you experienced dry mouth in the last six months?” or through multi-item approaches, such as the Xerostomia Inventory (XI) (1). The diagnosis of salivary gland hypofunction is based on salivary flow measurements. The term hyposalivation is used, when the unstimulated whole saliva secretion is ≤0.1 ml/min and the chew-stimulated ≤0.7 ml/min (9). Xerostomia and hyposalivation are common conditions, which often occur as side effects of numerous medications (10, 11). A systematic review by the World Workshop on Oral Medicine VI reported that nine of the 14 categories of medications are associated with xerostomia according to the first level of Anatomical Therapeutic Chemical (ATC) Classification (10). A recent survey in Sweden revealed a strong association between xerostomia and medication use, and a high prevalence of xerostomia (43.6%) among adults in primary care centers (12). Polypharmacy (i.e., the regular use of five or more medications at the same time) was particularly associated with a high prevalence of xerostomia (71.2%) among older adults (12). The reported prevalence of xerostomia in the Scandinavian literature varies widely, ranging from 0.9% to 64.8%, which is attributed to differences in measures used and the age of the populations studied (13, 14). Additionally, the prevalence of xerostomia is lower in men (10%–26%) than in women (10%–33%) (12, 15).

Five medication groups, including cardiovascular agents (C01–C10), analgesics (N02), psycholeptics (N05), antirheumatics (M01), and antithrombotics (B01) have been found strongly associated with a low saliva flow rate in a cross-sectional study conducted among older Caucasians aged 65 and above in Denmark (16). Similarly, a cohort study in Germany showed a reduction in stimulated saliva flow due to medications primarily affecting the cardiovascular (C) and nervous (N) systems (17). Another recent study found an association between cardiovascular medications and both xerostomia and increased caries activity in young adults (17). To date, a few studies have investigated the link between xerostomia and the use of different medication categories (15–19). As a result, there is a need for studies conducted in collaboration with healthcare providers to examine the association of xerostomia with different medication categories.

The primary objectives of this study are to: (1) determine the prevalence of xerostomia based on diagnosis made by healthcare providers in the region of Västra Götaland in Sweden, and to (2) investigate the association between xerostomia and medication categories, with a focus on the impact of the number of medications prescribed, while also considering factors such as age and sex. We hypothesize that the prevalence of xerostomia is high among adults and highly associated with intake of medications from different ATC categories, but it is likely that xerostomia is underrecognized by healthcare professionals.

## Materials and methods

This is an ecological retrospective study where the aggregated data was obtained from the regional administrative healthcare database in the region of Västra Götaland, Sweden (Vega). The data were extracted at the group level, without any personal information, from the Vega database with the assistance of statisticians from the Information Technology department in the region of Västra Götaland, Sweden. The study population consisted of the entire adult population (aged 18 years and above) registered with primary care providers in the Region of Västra Götaland, Sweden between 2019 and 2020. The population was stratified by age (18–35, 36–55, 56–70, and 71 + years) and sex (male and female). The exposure was intake of prescribed medication, while the outcome was a diagnosis of xerostomia, based on ICD-10 codes: R11.7 (disturbed salivary gland function) and R68.2 (unspecific dry mouth). Additionally, data were collected on patients who received an ICD-10 diagnosis code for Sjögren's disease (ICD-10: M35.0), as well as those diagnosed with head and neck cancers (ICD-10: C00–C14, C30–C32, C77.0), in combination with diagnosis codes R11.7 and R68.2.

Exposure was assessed in patients with and without the outcome by determining the number of patients using 0, 1, 2, 3, 4, and >5 medications, as well as the number of patients using medications from the following ATC categories (grouped according to the 1st level ATC classification): A (alimentary tract and metabolism), B (blood and blood-forming organs), C (cardiovascular system), D (dermatologicals), G (genitourinary and sex hormones), H (systemic hormonal preparations, excluding sex hormones and insulins), J (anti-infectives for systemic use), M (musculoskeletal system), N (nervous system), R (respiratory system), and no medications. Patients may also have a regular intake of multiple prescribed medications from different ATC groups. Therefore, those with multiple medications were counted multiple times, corresponding to the number of medications consumed. For patients diagnosed with xerostomia who were not using any medications, additional data on other assigned diagnosis was obtained from the Vega register. In the present study, patients were divided into xerostomia and non-xerostomia group according to the xerostomia diagnosis.

The Swedish Central Ethical Review Board approved the study (reg. no. 2020-03127). The study followed the ethical principles outlined in the Declaration of Helsinki and complies with relevant local and international standards for register-based research.

### Statistical analysis

The prevalence of xerostomia diagnosis according to the number and type of medications used was calculated using Microsoft Excel. The prevalence ratio of xerostomia diagnosis in patients taking more than one medication compared to those taking no medications was estimated using STATA 17 statistical software (20). This software was also used to determine the prevalence ratio (PR) of xerostomia diagnosis in patients taking medications categorized according to the ATC classification, compared to those not taking any medication. The odds ratio calculator from the STATA 17 epidemiology table was used to calculate the prevalence ratio and 95% confidence intervals (CI) (20). MedCalc's comparison of proportions calculator (21) was used to assess the significance of the prevalence of xerostomia by age and sex. Additionally, the prevalence of other diagnosis among patients with xerostomia who were not taking any medication was calculated using Microsoft Excel.

## Results

The study included data from 1,398,967 patients (733,325 females and 665,642 males). Among these, 3,254 patients had diagnosis of xerostomia (2,378 females and 876 males), with an overall xerostomia prevalence of 0.23%. A total of 1,395,713 patients (664,766 females and 730,947 males) were registered in the non-xerostomia group. A significantly higher proportion of patients diagnosed with xerostomia were using one or more prescribed medications than those without a xerostomia diagnosis (95.91% vs. 78.4%, respectively; *p* < 0.0001). Among the 3,245 patients diagnosed with xerostomia, 133 (4.09%) reported not using any medications. These patients had also reported symptoms (as registered in Vega database) including early satiety, decreased libido, fever without chills, and jaw pain. A total of 45 patients were diagnosed with both xerostomia and Sjögren's disease, of whom 6 had no prescribed medications. Additionally, 11 patients with xerostomia were diagnosed with cancer in the head and neck region, and 5 of these patients had no prescribed medications.

**Figure 1:**
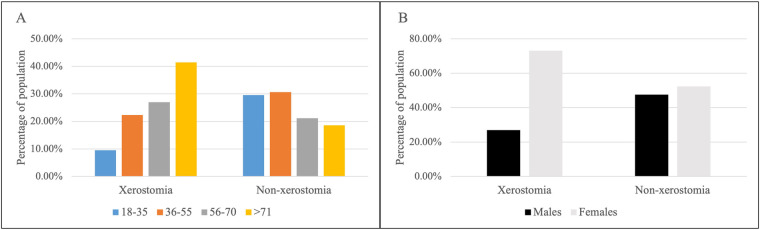
The percentage (%) of population with and without xerostomia diagnosis according to age group (18–35, 36–55, 56–70, and >71 years) **(A)** and sex (male or female) **(B)**.

Figure 1 displays the distribution of patients with and without a xerostomia diagnosis by age and sex. Among those diagnosed with xerostomia, females (73.08%) represented a significantly higher percentage compared to males (26.92%) (*p* < 0.0001). In contrast, the distribution of females (52.37%) and males (47.63%) without the diagnosis was similar.

Xerostomia diagnosis was lowest in the 18–35 years age group (9.56%) and highest in the >71 years age group (41.49%). The prevalence of the diagnosis correlated with advancing age. In contrast, the percentage of patients in the non-xerostomia group was similar for the 18–35 years (29.59%) and 36–55 years (30.56%) age groups, while the lowest percentage was observed in those aged >71 years (18.57%). The percentage of patients aged 55 years and above was significantly higher in the xerostomia group than in the non-xerostomia group (*p* < 0.0001).

The intake of medications and the xerostomia diagnosis were significantly associated across all medication categories listed in Table 1 (*p* < 0.0001). The highest percentage of patients diagnosed with xerostomia had medications in the categories of alimentary tract and metabolic diseases (16.07%), followed by diseases of the nervous system (16.05%), cardiovascular system (12.63%), and respiratory system (10.32%) (Table 1).

**Table 1:** Medication intake categorized according to the ATC classification in the two groups with and without xerostomia. A significantly higher (*p* < 0.0001) proportion of patients taking one or more prescribed medications were diagnosed with xerostomia.

ATC classification	Xerostomia		Non-xerostomia		*p*-value
A: Alimentary tract and metabolism	2,287	16.07%	462,397	12.14%	<0.0001
B: Blood and blood forming organs	1,317	9.26%	284,574	7.47%	<0.0001
C: Cardiovascular system	1,797	12.63%	428,715	11.25%	<0.0001
D: Dermatologicals	964	6.78%	251,407	6.60%	<0.0001
G: Genitourinary system and sex hormones	1,012	7.11%	285,456	7.49%	<0.0001
H: Systemic hormonal preparations, excluding sex hormones and insulins	821	5.77%	190,951	5.01%	<0.0001
J: Anti-infectives for systemic use	1,130	7.94%	389,815	10.23%	<0.0001
M: Musculoskeletal system	1.015	7.13%	298,401	7.83%	<0.0001
N: Nervous system	2,283	16.05%	542,684	14.24%	<0.0001
R: Respiratory system	1,469	10.32%	373,926	9.81%	<0.0001
No medications	133	0.93%	301,509	7.91%	<0.0001

**Figure 2:**
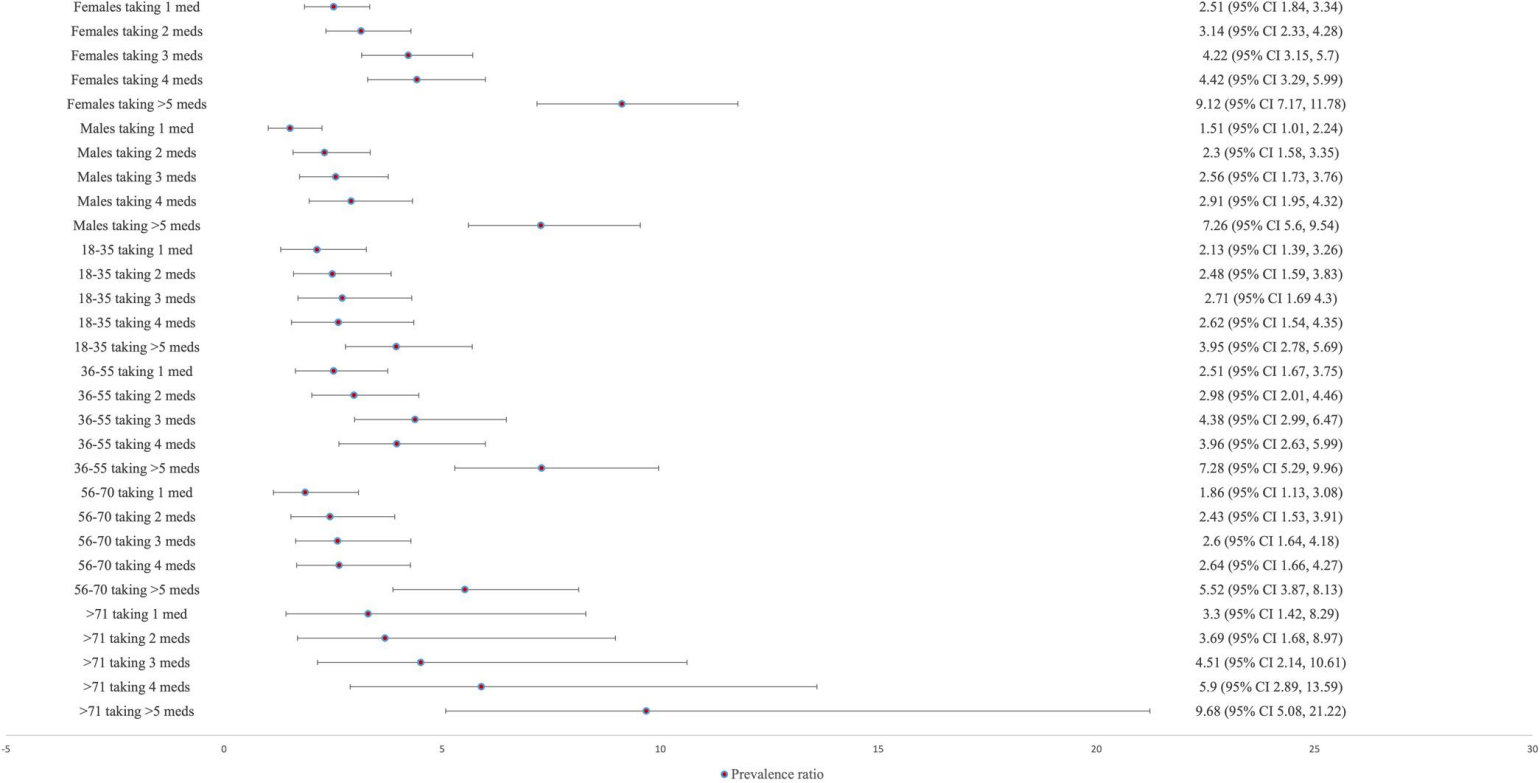
The prevalence ratio of xerostomia based on sex (male, female), age groups (18–35, 36–55, 56–70, and 71+), and number of medications (1 medication, 2–4 medications, ≥5 medications). Xerostomia diagnosis shows highest prevalence with intake of more than 5 different medications (polypharmacy) in comparison to no intake of medications, irrespective of age and sex.

Figure 2 shows the prevalence ratio of xerostomia in relation to the number of medications. Patients taking one or more medications were more likely to be diagnosed with xerostomia than those not taking any medications, irrespective of age and sex. In the case of polypharmacy, the prevalence of xerostomia diagnosis was 3.95 times higher in the 18–35 years age group (95% CI 2.78–5.69, *p* < 0.0001), 7.28 times higher in the 36–55 years age group (95% CI 5.29–9.96, *p* < 0.0001), and 5.52 times higher in the 56–70 years age group (95% CI 3.87–8.13, *p* < 0.0001) compared to patients with no medication intake. A higher percentage of males (26.78%) than females (16.83%) were unmedicated. An intake of three or more medications was higher in females (62.88%) than in males (50.27%). Patients with polypharmacy and above the age of 71 years had a 9.68 times higher prevalence of xerostomia diagnosis (95% CI 5.08–21.22, *p* < 0.0001) than younger patients.

**Figure 3:**
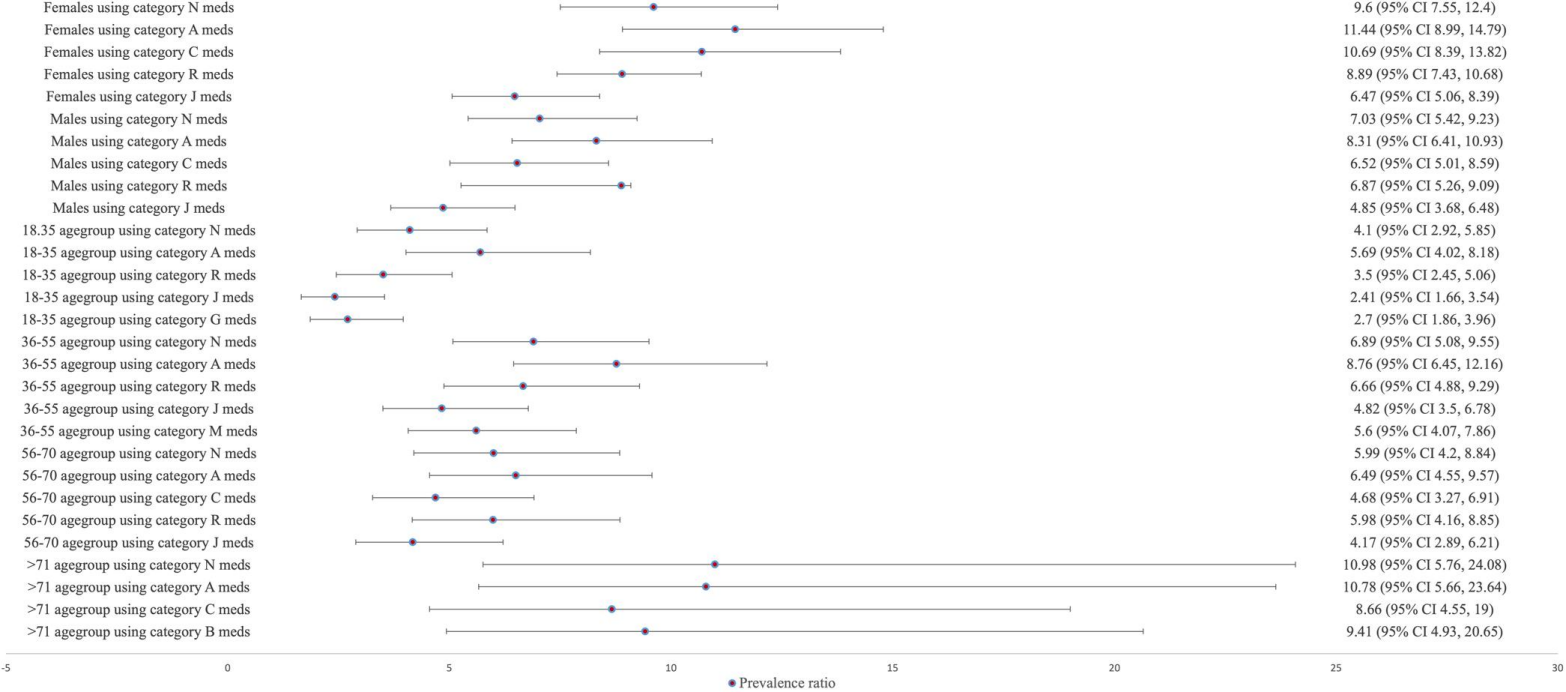
The prevalence ratio of xerostomia according to sex (male, female), age groups (18–35, 36–55, 56–70, and 71+), and medication categories based on the ATC classification. In comparison to those patients not taking any prescribed medication, xerostomia diagnosis shows highest prevalence in those consuming ATC category A (alimentary tract and metabolism) medications, irrespective of sex and age. Only those aged above 71 years had the highest prevalence of xerostomia among those taking ATC category N (nervous system) in comparison to those not taking any prescribed medication.

Figure 3 shows the prevalence ratio of xerostomia diagnosis according to the type of medications used. In females taking medications from the alimentary tract and metabolism category, the prevalence of xerostomia was 11.44 times higher (95% CI 8.99–14.79) than in females not taking any medications. This was followed by medication categories from the cardiovascular system (PR: 10.69; 95% CI 8.39–13.82), the nervous system (PR: 9.6; 95% CI 7.55–12.4), the respiratory system (PR: 8.89; 95% CI 7.43–10.68), and systemic anti-infectives (PR: 6.47; 95% CI 5.06–8.39). Similarly, among males taking medications from the alimentary tract and metabolism category, the prevalence of xerostomia was 8.31 times higher (95% CI 6.41–10.93) than in males not taking any medications. This was followed by medication categories from the nervous system (PR: 7.03; 95% CI 5.42–9.23), the respiratory system (PR: 6.87; 95% CI 5.26–9.09), the cardiovascular system (PR: 6.52; 95% CI 5.01–8.59), and the systemic anti-infectives (PR: 4.85; 95% CI 3.68–6.48).

Regarding age, intake of medications from the alimentary tract and metabolism category were associated with the highest prevalence of xerostomia diagnosis, with 5.69 times (95% CI 4.02–8.18) higher in the 18–35 age group, 8.76 times (95% CI 6.45–12.16) in the 36–55 age group, and 6.49 times (95% CI 4.55–9.57) in the 56–70 age group, compared to those not taking any medications. In the age group over 71 years, medication category from the nervous system had the highest prevalence of xerostomia diagnosis (10.98 times higher, 95% CI 5.76–24.08) compared to those not taking any medications.

## Discussion

This study showed that 0.23% of a population of patients in primary care centers in the Västra Götaland region was diagnosed with xerostomia. The prevalence was higher in females (about 73%) than in males (about 27%). Xerostomia was most prevalent in patients over 71 years of age (about 42%), and least prevalent in those aged 18–35 years (about 10%). The medications in the categories for the alimentary tract and metabolism (about 16%), and the nervous system (about 16%) were significantly associated with the diagnosis of xerostomia in both sexes, but with a stronger association observed in females. Additionally, medication categories for cardiovascular system (about 13%), respiratory system (10%), and anti-infective agents (8%) were also associated with a high prevalence ratio of xerostomia. Across most age groups, medications classified under the alimentary tract and metabolism (ATC class A) category showed the strongest association with xerostomia, except in patients over 71 years of age, where medication intake from nervous system category revealed the strongest association.

ATC code A includes a large variations of agents used for treating gastrointestinal disorders (e.g., antispasmodics, anti-diarrheals), antiemetics and antinauseants, agents for bile and liver therapy, anti-inflammatory agents (e.g., loperamide, rifaximin), anti-obesity preparations (e.g., orlistat, liraglutide), antidiabetic agents (e.g., insulin, metformin, sulfonylureas, SGLT-2 inhibitors), appetite stimulants, and minerals and vitamins. Many of these medications can directly or indirectly interfere with the mechanisms of salivary secretion and can lead to xerostomia and/or salivary gland hypofunction. For example, antiemetics and antinauseants can impact salivary secretion, mainly by inhibition of parasympathetic and sympathetic signaling pathways (22). Similarly, antispasmodics typically inhibit parasympathetic signaling (cholinergic signaling) in the acinar cells via muscarinic receptors (22). Anti-diarrheals, particularly those containing opioid derivatives (like loperamide or diphenoxylate), can also inhibit salivary secretion both centrally and peripherally through anticholinergic actions and thereby diminishing parasympathetic signaling (22). Accordingly, the findings of our study support the previous studies showing that medications interfering with the autonomic nervous system and acting on parasympathetic and sympathetic receptors, are often associated with xerostomia due impaired salivary secretion (11, 23). Additionally, medications in ATC class A for treating liver diseases like hepatitis (e.g., interferons, ribavirin) can affect fluid balance and may lead to xerostomia without effect on nerve signaling (24). This can occur due to gastrointestinal side effects and dehydration, which may indirectly reduce saliva secretion.

The plasma membranes of salivary gland cells contain muscarinic cholinergic and adrenergic (α1 and β1) receptors that control secretion. Unlike other organs, parasympathetic and sympathetic activity in the salivary glands work synergistically through shared intracellular signaling pathways and neuropeptides (e.g., calcitonin gene-related peptide, vasoactive intestinal peptide, substance P, nitric oxide) to enhance fluid and protein secretion (25). Blocking one receptor type disrupts both its response and the synergistic effect of the others (26). Consequently, intake of several medications is likely to induce xerostomia. Our study revealed a significant association between the prevalence of xerostomia and the number of medications taken on daily basis, especially polypharmacy, and corroborate findings of previous studies (17, 26, 27). The likelihood of xerostomia is also associated with age (above 71 years), where polypharmacy is most prevalent. Global life expectancy has increased, particularly in developed nations, leading to an aging population and a rise in multimorbidity and polypharmacy (28). Polypharmacy, defined as the regular intake of five or more different medications, has become a significant health concern, with its prevalence in Sweden rising from 3.8% in 2006 to 5.1% in 2014 (29). The rising prevalence of polypharmacy poses challenges due to the risks of drug interactions and side effects. Supporting earlier findings, Adolfsson et al. reported that 71.2% of patients in primary care centers, mostly women, had xerostomia due to polypharmacy (12). These findings highlight the importance of healthcare providers being aware of the side effects of medications, especially in the cases of polypharmacy, and to effectively managing these patients by relieving symptoms and preventing the consequences of hyposalivation.

Although the study emphasizes the strong association between xerostomia and medication use, it also shows that 133 patients (4.09%) diagnosed with xerostomia had not been prescribed any medication. This subgroup highlights the importance of considering other causes of xerostomia, including Sjögren's disease, viral infections, radiation therapy for head and neck cancers, and other systemic conditions. A recent study by Ghalwash et al. highlights the need to raise clinical awareness regarding the symptoms of dry mouth and its associated systemic manifestations, particularly in the context of diagnosing and managing Sjögren's disease, which remains significantly underdiagnosed (30).

In this study, the female gender, polypharmacy and age above 71 years were associated with the highest prevalence of xerostomia. A higher percentage of males (about 27%) than females (about 17%) were unmedicated. Conversely, a higher percentage of females (about 63%) than males (about 50%) had a regular intake of three or more medications. This is in line with previous studies indicating a higher prevalence of polypharmacy among females (10, 12). Women are more likely to seek the healthcare system when have symptoms, they have a larger intake of medications from the genitourinary system and sex hormones category, and they have a longer life expectancy than males, which can explain this gender difference (31–33). Our study supported a larger percentage of women take medications from the genitourinary system and sex hormones category with the highest intake in the age group 18–35 years and 36–55 years. As regard to the sex differences, the current study found women in the xerostomia group had a higher prevalence of medications affecting the nervous system, whereas men more commonly used medications targeting the alimentary tract and cardiovascular system. This aligns with findings by Smidt et al. and Laugisch et al., as cardiovascular conditions are more prevalent in men (16, 17), while nervous system disorders are more common in women, according to the WHO (34). These differences may also explain the sex-based variation in medication use.

The prevalence of patients diagnosed with xerostomia was surprisingly low in our study and contrasts the prevalence of 43.6% reported among patients registered in primary care settings in the same region of Sweden (12). This discrepancy, along with the overall low rate of xerostomia diagnosis in primary healthcare, is notable, especially given the high prevalence of the condition. Though there are differences in the study design, the current study has a population- based register design while the previous study used an individual-based cross-sectional design. The findings of our study suggest that xerostomia and registration of the diagnosis may be overlooked by healthcare professionals. This may also reflect a clinical recognition gap, where symptoms like dry mouth are not adequately explored or documented during consultations. Improving awareness and diagnostic precision could help ensure that underlying conditions, whether pharmacological or non-pharmacological, such as Sjögren's disease, are more consistently identified and assigned appropriate clinical codes.

The current study has an advantage to include a broad population of patients to reveal the association between medication types and xerostomia diagnosis in primary care settings. Previous studies had limitations with insufficient sample size to assess the impact of different medication categories and risk factors on xerostomia (10). A recent, larger cohort study of xerostomia patients from dental clinics, predominantly females, observed the association between diseases, medications, and their impact on saliva flow rates (19). The study could not provide results for all medication categories due to the limited number of patients in each medication category. However, patients taking antidepressant medications affecting the nervous system had lower saliva flow rates compared to those with depression who were not using antidepressants, highlighting the key role of medications in salivary secretion in patients with depression (19). In the present study, patients using medications targeting the nervous system, cardiovascular system, respiratory system, alimentary system, blood-forming systems, and systemic anti-infectives were strongly associated with xerostomia, regardless of age and sex. This is consistent with the results of a systematic review that found a strong association between dry mouth and medications affecting the nervous system, respiratory system, musculoskeletal system, and cardiovascular system, with a moderate association observed for systemic anti-infectives, all of which are linked to salivary gland hypofunction (10). It is possible that patients who did not take any prescribed medications, were taking over-the-counter drugs or had undiagnosed conditions affecting the salivary glands at the time. A key limitation of this study is that the registry data included only prescribed medications, thereby excluding information on over-the-counter medication use. Consequently, the actual burden of xerostomia may be higher than reported, particularly among patients using over-the-counter medications known to cause dry mouth.

## Conclusion

This population-based study identified a low recorded prevalence of xerostomia (0.23%) in primary care settings, despite strong associations with medication use, age, and sex. Xerostomia was most prevalent among women and individuals aged over 71 years, with polypharmacy linked to a 9.68-fold increase in prevalence. Medications targeting the nervous, cardiovascular, and respiratory systems, along with systemic anti-infectives, were particularly associated with xerostomia. Despite the high burden of xerostomia, these findings highlight the need for heightened clinical awareness and support routine diagnostic assessment in primary care. Future research may focus on exploring the clinical recognition gap between patients’ subjective symptoms and clinical diagnoses through the integration of registry data. It could also focus on developing simple diagnostic tools to aid in identifying xerostomia and distinguishing between pharmacological and non-pharmacological systemic causes, including conditions such as Sjögren's disease. Advancing these efforts may improve diagnostic accuracy and enable more targeted management strategies in clinical practice.

## Data Availability

All relevant data is contained within the article: The original contributions presented in the study are included in the supplementary material, further inquiries can be directed to the corresponding author.

## Supplementary material

Supplementary Figure 1 The prevalence of other diagnosis in patients with xerostomia and without medications showed that 96.99% were diagnosed under R68 (Other general symptoms and signs), followed by Z71 (Counseling and medical advice, not elsewhere classified) at 30.83%, R53 (Malaise and fatigue) at 19.55%, and R52 (Pain, not elsewhere classified) at 17.29%.
