# Supplementary figures and images for: Xerostomia in primary care: a register-based study of prevalence, medication categories, and associated risk factors
Source: Front Oral Health. 2025 Oct 10;6:1684568. doi: 10.3389/froh.2025.1684568 (PMC12549705; doi:10.3389/froh.2025.1684568)

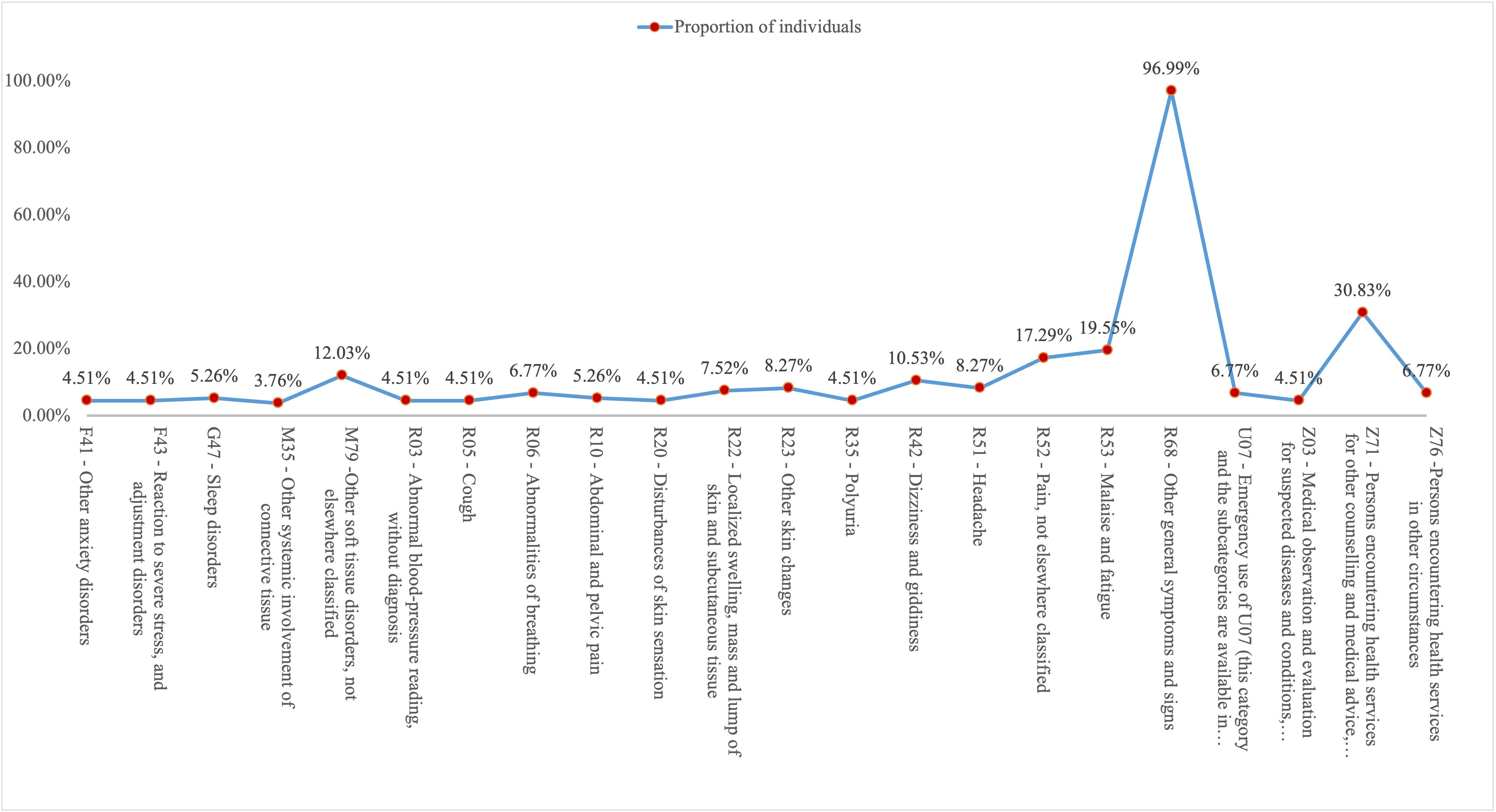

Supplement: Supplementary Figure 1 — The prevalence of other diagnosis in patients with xerostomia and without medications showed that 96.99% were diagnosed under R68 (Other general symptoms and signs), followed by Z71 (Counseling and medical advice, not elsewhere classified) at 30.83%, R53 (Malaise and fatigue) at 19.55%, and R52 (Pain, not elsewhere classified) at 17.29%. [file Image1.jpeg]
